# Stakeholder Roles and Views in the Implementation of the Differentiated HIV Treatment Service Delivery Model Among Female Sex Workers in Gauteng Province, South Africa

**DOI:** 10.3390/healthcare13182329

**Published:** 2025-09-17

**Authors:** Lifutso Motsieloa, Edith Phalane, Refilwe N. Phaswana-Mafuya

**Affiliations:** South African Medical Research Council/University of Johannesburg Pan African Centre for Epidemics Research Extramural Unit, Faculty of Health Sciences, University of Johannesburg, Johannesburg 2092, South Africa; edithp@uj.ac.za (E.P.); refilwep@uj.ac.za (R.N.P.-M.)

**Keywords:** female sex workers, key populations, HIV treatment, differentiated service delivery model, stakeholders, South Africa

## Abstract

**Background:** Key populations (KPs), particularly female sex workers (FSWs), continue to face significant barriers in accessing HIV-related healthcare services in South Africa. Structural challenges have historically hindered equitable HIV treatment access, worsened by the COVID-19 pandemic. Overburdened clinics, staff shortages, and travel constraints disrupted HIV services and ART adherence. In response, the Differentiated Service Delivery (DSD) model was rapidly scaled up to decentralise care and improve treatment continuity. **Objective**: To solicit the views of stakeholders regarding their interests, roles and experiences in the implementation of the HIV treatment DSD model among FSWs in South Africa, as well as associated successes and barriers thereof. **Methods**: We purposively selected and interviewed eight stakeholders, comprising government officials, implementers and sex workers’ advocacy organizations. Thematic analysis was used to explore the perceived impact of DSD models and associated successes and barriers in the current service delivery landscape. **Results**: The study found that decentralization of DSD models improved access to services for FSWs. However, the criminalization of sex work perpetuates fear and marginalization, while stigma and discrimination within healthcare settings remain significant deterrents to HIV treatment uptake. High mobility among FSWs also disrupts continuity of care, contributing to treatment interruptions and lack of data on loss to follow-up. Participants highlighted the need for legal reform, increased healthcare provider sensitization, and the integration of mental health and psychosocial support in HIV services. Peer-led interventions and digital health innovations, such as biometric systems and electronic medical records, emerged as promising strategies for enhancing patient tracking and retention. Nonetheless, the sustainability of DSD models is threatened by an overreliance on external donor funding and insufficient government ownership. **Conclusions**: To achieve equitable healthcare access and improved HIV outcomes for KPs, especially FSWs, a multi-pronged, rights-based approach is essential. This must include community engagement, structural and legal reforms, integrated support services, and sustainable financing mechanisms to ensure the long-term impact and scalability of DSD models.

## 1. Introduction

South Africa remains the epicenter of the global Human Immunodeficiency Virus (HIV) pandemic, and home to the largest population of people living with HIV (PLHIV) worldwide [1]. As of 2023, there were approximately 8.3 million PLHIV in the country, of whom 5.9 million were receiving antiretroviral therapy (ART) [1,2]. While South Africa has made notable strides in HIV testing, achieving a diagnosis rate of 95.4%, treatment coverage is lagging at 78.7% of PLHIV on ART. Despite an encouraging viral suppression rate of 91.3% among those on ART, the fact that one in four PLHIV remain untreated highlights a critical gap in achieving the UNAIDS 95-95-95 targets to be reached by 2030 [3]. This treatment gap, particularly among key populations (KPs), remains a major barrier to controlling the HIV epidemic in South Africa [4].

Structural barriers within South Africa’s public healthcare system have long impeded consistent and equitable access to HIV treatment services [5]. The existing criminalization policies against the FSWs have created significant barriers to accessing HIV services, particularly for outreach and community-based Differentiated Service Delivery (DSD) models. According to the literature, criminalization exposes FSWs to police violence, harassment, arbitrary arrests, and stigma, all of which reduce engagement in essential health services, consequently disrupting continuity of care [6]. These challenges reduce the success of DSD interventions that rely on safe, stigma-free environments and strong peer-led engagement. Evidence from countries such as New Zealand, where sex work has been decriminalized, shows improved health outcomes, reduced violence, and strengthened access to HIV services without increasing human trafficking outcomes that directly support DSD implementation [7]. Regional advocacy organizations, such as the African Sex Workers Alliance (ASWA), Sex Workers Education and Advocacy Taskforce (SWEAT), and the Asijiki Coalition, continue to advocate for the decriminalization of sex work, highlighting the importance of legal reform in realizing the full potential of differentiated, client-centered HIV care [8]. In this context, decriminalization is not only a human rights imperative, but also a structural enabler necessary for the long-term viability and scalability of DSD models in advancing HIV care for the key populations especially FSWs.

Overcrowded clinics, staff shortages, long queues, and inconsistent access to treatment have characterized many public health facilities in South Africa [9]. These systemic challenges, which were already significant before the COVID-19 pandemic, were exacerbated during the crisis created by the pandemic. Lockdowns that were accompanied by movement restrictions led to widespread disruptions in HIV testing, treatment initiation, and ART refill services [10]. Many PLHIV missed appointments, not due to unwillingness, but because services were inaccessible, often requiring long and costly journeys to health facilities [11]. This disproportionately affected KPs, who had to contend with additional barriers, including stigmatization and discrimination [12]. In response, South Africa rapidly expanded its implementation of the DSD models to sustain and improve access to HIV treatment during and after the COVID-19 pandemic [13]. By decentralizing service delivery and shifting routine care away from overburdened clinics, the DSD models were aimed at improving retention of care, reducing missed appointments, and addressing logistical barriers that previously undermined ART adherence. Decentralization refers to the intentional transfer of authority, resources and decision-making from central governments to lower levels of the health system, such as districts or community-based structures. It is often pursued as a governance strategy to improve service responsiveness, promote equity, and ensure that healthcare delivery is more aligned with local contexts and needs [14]. Importantly, decentralization is not merely a reaction to dysfunction but a proactive and strategic intervention that can help resolve systemic inefficiencies.

By contrast, a fragmented health system is characterised by poorly coordinated services, weak integration across levels of care, and disjointed delivery platforms. Such fragmentation often leads to service duplication, care discontinuities, and reduced accessespecially for key populations. While decentralization may emerge as a response to such fragmentation, the two concepts are not inherently linked. Instead, decentralization can serve as a corrective mechanism to address fragmentation by promoting integration and improving governance at the point of service delivery.

In the context of DSD for HIV, decentralization enhances the model’s potential for sustainability and scale. When DSD is incorporated into national strategies, supported by domestic financing, and integrated within existing health systems, it can strengthen continuity of care and institutional resilience. However, when HIV services including DSD models are implemented primarily through donor-driven projects that operate in parallel to national systems, it risks becoming a temporary fix that addresses immediate service gaps without contributing to long-term health system strengthening [15,16].

Differentiated Service Delivery models are client-centered approach to HIV care that seeks to simplify and modify health services to better suit the different needs of individuals living with and at risk of HIV, while also increasing health system efficiency [17]. The World Health Organisation (WHO) first publicly adopted the DSD models in 2016, in response to the awareness that traditional, one-size-fits-all methods were insufficient for achieving equitable access, retention, and treatment results, particularly among underprivileged groups [4]. The DSD models differ in design, but they usually incorporate changes to the “*when*”, “*where*”, “*who*”, and “*what*” of service delivery, such as allowing for shorter visits, decentralizing medicine administration, and incorporating peer support and community-based care [17].

In South Africa, FSWs are disproportionately impacted by HIV, with rates much higher than the overall population owing to a combination of biological, social, economic, and legal vulnerabilities [18,19]. Historically marginalized and criminalized, FSWs frequently work in circumstances marked by poverty, gender-based violence, mobility, stigmatization, and exclusion from official health systems [20,21]. These structural impediments limit access to consistent HIV prevention and treatment programs, increase risk exposure, and fuel distrust in traditional healthcare settings.

Recognizing these complex challenges, the DSD models designed for FSWs seek not just to enhance clinical results but also to promote dignity, agency, and responsiveness within HIV programs [4,22]. Community-led efforts, peer navigation, mobile outreach, and multi-month dispensing are increasingly being used in such models, with the intention of addressing both the medical and social realities that shape FSWs’ health-seeking behaviors [23].

The successful rollout of the DSD models in South Africa has been underpinned by collaborative multisectoral efforts. The National Department of Health (NDoH) played a central role in providing policy leadership and ensuring that DSD strategies are integrated into national health priorities [13]. Frontline healthcare workers: nurses, doctors, and clinic managers have offered critical feedback to ensure that the DSD models are clinically feasible and responsive to patient needs [24,25]. Community-based organizations (CBOs) and non-governmental organizations (NGOs) were also instrumental in adapting DSD services to reach underserved and high-risk populations and worked hard to address structural and social barriers, ensuring that these groups are meaningfully included in DSD implementation [10,26]. Equally significant is the involvement of PLHIV themselves. Advocacy groups, such as the Treatment Action Campaign (TAC) and the South African National AIDS Council (SANAC), provided platforms for PLHIV to share their experiences and influence the design and delivery of DSD services [27]. These lived experiences helped shape responsive DSD models, not just to clinical needs, but also to social realities, and the improvement of service acceptability and uptake.

International donors, such as the U.S. President’s Emergency Plan for AIDS Relief (PEPFAR) and the Global Fund (GF), provided essential financial and technical resources. Their support enabled the piloting, scaling, and ongoing refinement of DSD models initiatives in South Africa [28]. However, the recent geopolitical situation resulting in the cessation of PEPFAR funding is threatening continued support [29]. Private sector partners, such as pharmacies and health insurers, have also played an increasingly important role by providing expanded ART pick-up points and thereby reducing reliance on public healthcare facilities [10].

The development and scale-up of the DSD models in South Africa illustrate the value of coordinated stakeholder sector-driven approaches in addressing complex healthcare delivery challenges. However, understanding how various stakeholders perceive their roles, experiences, successes and barriers in the implementation of the DSD model remains critical for informing future policy and practice. Exploring these perspectives provides actionable insights to strengthen the DSD models, ensure sustainability, and advance South Africa’s goal of universal HIV treatment coverage.

This study aimed to solicit the views of stakeholders regarding their interests, roles and experiences in the implementation of the HIV treatment DSD model among FSWs in South Africa, as well as associated successes and barriers thereof.

## 2. Materials and Methods

### 2.1. Study Design and Setting

A qualitative design was used to describe the views of stakeholders regarding their interests, roles and experiences in the implementation of the HIV treatment DSD models among FSWs in South Africa, as well as associated successes and barriers. This was done, firstly, by conducting an analysis and mapping of stakeholders, followed by semi-structured interviews with the stakeholders to elicit their views. This qualitative approach provided a deeper explanation of the practical realities and lived experiences of those involved in the implementation and design of the DSD models, offering valuable insights to inform future strategies for strengthening service delivery [30]. The study was conducted in the Gauteng province of South Africa. Gauteng is the smallest of South Africa’s provinces, covering an area of 18,178 km^2^ or approximately 1.4% of the total surface area of South Africa. As the most populous province, hosting approximately 15.93 million residents, it represents 25.3% of the nation’s population [31]. The province has the highest burden of HIV with a prevalence of 11.4% [3]. Female Sex Workers’ prevalence in the province remains high at 47.8% [3]. The concentration of urban centres in Gauteng, along with associated socio-economic factors, contributes to a higher presence of FSWs [32]. The detailed methodology for this work has been published [33].

### 2.2. Methods for Stakeholder Analysis and Mapping

An analysis and mapping of stakeholders was conducted to identify, prioritize, and engage key stakeholders involved in the implementation of the HIV treatment DSD models among FSWs in South Africa.

*The stakeholder analysis and mapping focused on four areas:*

Stage One: Identification of stakeholders engaged in DSD implementation.
Stage Two: Understanding the interests, roles, and experiences of the stakeholders.
Stage Three: Assessing the influence on DSD outcomes.
Stage Four: Developing engagement strategies to gather detailed insights.

Stage One: Identification of Key Stakeholders

The snowball sampling technique was used to identify key stakeholders working with FSWs in the Gauteng Province, South Africa. Stakeholders were recruited through collaboration with HIV implementing partners, who connected the researcher with gatekeepers and program managers to facilitate access. Gatekeepers helped identify potential participants and distributed study information letters outlining the study’s objectives, procedures, and confidentiality measures. The sample included representatives from National and Provincial government health departments, NGOs and community-based organizations (CBOs), civil society organizations (CSOs), implementing partners, UN agencies and multilateral donors, such as PEPFAR and the Global Fund. This approach ensured diverse representation across the HIV treatment, care, and support continuum.

Stage Two: Understanding Stakeholders’ Roles and Experiences

After identification, stakeholders were profiled to identify their interests, roles, and experiences in DSD implementation. Data collection focused on their contributions to initiatives such as community ART refill groups, adherence clubs, and multi-month dispensing. Stakeholders also shared barriers, successes, and recommendations for improving DSD delivery service.

Stage Three: Assessment of Stakeholders’ Influence and Impact

Stakeholders were assessed for their level of influence and interest, using a four-quadrant matrix. The matrix is categorized into High Interest, High Influence (Key Players); High Influence, Low Interest (Keep Satisfied); Low Influence, High Interest (Keep Informed); and Low Influence, Low Interest (Monitor). Those with high interest and high influence were prioritized for in-depth engagement. This mapping clarified stakeholders who had the greatest impact on DSD implementation and whose insights were critical for evaluating program outcomes and informing improvements.

Stage Four: Stakeholder Engagement Strategies

Based on influence-interest mapping, tailored engagement strategies were developed. These included targeted meetings, consultations, and semi-structured interviews, along with ongoing communication to facilitate continuous feedback. Engagement activities aimed to encourage open dialogue about the DSD models successes, barriers, and areas needing adjustment [34].

#### Methods for In-Depth Interviews with Stakeholders

The study explored stakeholders’ perspectives on their roles, interests, successes, barriers, and recommendations for improving DSD implementation.

### 2.3. Study Participants

The study participants were selected from a list of stakeholders obtained through stakeholder analysis and mapping. A purposive sample of eight stakeholders aged 18 and above was recruited to obtain rich detailed information on the HIV treatment DSD models for FSWs. Stakeholders included policymakers, executives, coordinators, supervisors, or managers, advising, informative, and advocacy roles, FSWs, and PLHIV participating in HIV prevention, treatment, care, and other DSD model-supporting interventions.

### 2.4. Recruitment of Participants

Participants were identified through Stage One of the stakeholder analysis and mapping and selected to take part in the study. In person information sessions about the study were held with participants and shared via email with those unable to attend in person. Stakeholders who expressed interest contacted the researcher directly to schedule interviews at a convenient time and location. This recruitment approach leveraged trusted networks and promoted voluntary participation, ensuring a diverse range of perspectives among stakeholders.

### 2.5. Data Collection

Face-to-face interviews were conducted with the key stakeholders in spaces that were convenient and safe. A semi-structured interview guide using a questionnaire was used to explore stakeholders’ perspectives on DSD model initiatives, service structures, policies, and the challenges encountered, particularly in addressing priority needs, service gaps, and opportunities for improving HIV care for FSWs. Each semi-structured interview lasted around 30 and 60 min per participant. The interviews were conducted between 11 November 2024 and 12 March 2025. Stakeholders submitted filled informed consent forms before taking part in the study. Additionally, written permission was obtained from each participant for the interviews to be audio-recorded. Stakeholders were interviewed in English. Field notes were taken to supplement audio recordings, thereby enhancing data reliability and ensuring a thorough capture of information. The interviews continued until data saturation, which refers to the point where there was no new or substantial information emerging [35].

During the data collection the stakeholders were assured that their participation was strictly voluntary. Moreover, stakeholders’ privacy and confidentiality were assured, using pseudonyms and interview transcripts that did not contain any personally identifiable information. To ensure privacy and confidentiality, all data were de-identified by assigning pseudonyms, such as “Stakeholder 1”, to the participants, as opposed to using their names. Furthermore, all the data collection research materials and all collected data were stored securely. Electronic files were protected by password access on a computer, while physical documents, including signed consent forms, were kept in a locked location. Audio recordings will be deleted two years after the publication of the study, or after six years, if the study remains unpublished.

### 2.6. Data Analysis

The study employed a grounded, inductive approach to qualitative analysis. Interview recordings were manually transcribed verbatim in English, eliminating the need for translation. The transcripts were imported into ATLAS.ti version 22 (Scientific Software Development GmbH, Berlin, Germany) for systematic arrangement and analysis without predetermined codes or theoretical frameworks to allow patterns to emerge organically from the data [36]. The analytic process commenced with open coding, where text segments were carefully examined and tagged with descriptive labels emerging directly from the data. Through constant comparative analysis, these initial codes were refined, grouped into meaningful categories, and developed into broader thematic structures. A thematic content analysis was conducted collaboratively between the researcher and an external coder using ATLAS.ti’s inter-coder agreement function to compare coding decisions and resolve discrepancies, thereby enhancing the reliability of the coding process [36]. Analytical memos documented the evolving conceptual framework and decision trail throughout the analysis. Network visualizations were employed to explore relationships between emergent themes, while rigorous peer debriefing ensured interpretive validity. This systematic approach culminated in theoretically sensitive findings grounded in the data, with comprehensive reports generated to trace the analytic journey from raw transcripts to substantive conclusions.

### 2.7. Ethical Considerations

This study has been approved by the University of Johannesburg (UJ) Higher Degrees Committee and Research Ethics Committee (REC-2519-2024). Gatekeeper approvals were obtained from the Gauteng Province: Sedibeng District Health Research Committee (GP_20245_006) and AIDS Foundation of South Africa (AFSA), before data collection. During the recruitment stage, stakeholders received a research information letter that detailed all the information on the study before they completed the written informed consent forms. In addition, stakeholders were informed that their involvement in the data gathering was entirely optional.

## 3. Results

### 3.1. Characteristics of Participants

A total of eight stakeholders (six females and two males), aged between 30 and 57 years and predominantly Black African, participated in the study. Each stakeholder plays a critical role in the implementation of the DSD models in South Africa and, on average, had 9.62 years of work experience. The highest level of education among participants was a master’s degree.

### 3.2. Stakeholder Analysis

Table 1 outlines the roles and influence levels of the stakeholders. Government departments and United Nations (UN) agencies played a high-impact role, primarily through policy development, implementation, and technical support. Sex worker advocacy groups contribute significantly through leadership and advocacy, with a medium-to-high impact. Implementing partners focused on both advocacy and program implementation, exerting a medium level of influence. This distribution highlights the need for coordinated efforts across different sectors to ensure effective and inclusive DSD strategies. This also underscores the importance of tailored engagement strategies, with active collaboration focused on key stakeholders and strategic communication with peripheral but influential partners.

### 3.3. Results from the Stakeholders’ Views on the Implementation of the Differentiated Service Delivery Model

Four main themes were identified. These included (1) health care delivery and service model, (2) sustainability and innovation in health care systems, (3) structural and systematic barriers to health care access, and (4) facilitators associated with the implementation of the DSD model.

Theme 1. Health care delivery and service model

The effectiveness of healthcare interventions depends on integrated service models, prevention strategies, and tailored care approaches.

Sub-theme 1.1: HIV Prevention and Treatment

The integration of prevention and treatment services within community settings increases accessibility and broadens the reach of interventions. These holistic approaches address the broader health needs of both FSWs and the general population. Community-based and peer-led DSD models have enhanced access to HIV prevention and treatment services among KPs, especially FSWs. Pre-exposure prophylaxis, ART, and STI services are increasingly delivered through mobile clinics and outreach programs.

*“We have seen also that the viral loads were very high to the majority of the sex workers when we started, then overtime using case management service sex workers viral load decreased and majority were virally suppressed ….”*

(Stakeholder 8)

*“Its (DSD) preventing new infections and improves the health outcomes for people living with HIV…ensuring there is a provision of PrEP services and all the HIV interventions like your testing, services for HIV, your STIs, your condom use too.”*

(Stakeholder 5)

Sub-theme 1.2: Integration of Health Services

An integrated approach that combines clinical care with psychosocial support improves health outcomes and engagement in treatment. Integrating mental health and psychosocial support by social workers into HIV care enhances adherence and reduces viral loads. This comprehensive service delivery addresses the multifaceted needs of FSWs, ensuring sustained HIV care engagement.

*“I think HIV program or DSD model should also involve mental health so that clients can be motivated to continue to be on ARVs...”*

(Stakeholder 5)

*“We have seen also that the viral loads were very high to the majority of the sex workers when we started, then we provided services where I included the social workers then we saw the viral load decreasing and majority of sex workers are on treatment and virally suppressed.”*

(Stakeholder 8)

Sub-theme 1.3: Sensitization and Capacity

Building sensitization efforts have improved service delivery, but gaps remain, particularly in funding. Sustained investment in training, sensitization, and government collaboration is essential to institutionalizing non-discriminatory practices in health care. While sensitization efforts have contributed to a gradual reduction in stigma, deeper policy integration and systemic ownership are needed for lasting impact

*“A lot more funding needs to be injected into stigma, discrimination, sensitizing, and training of police and healthcare workers. There also needs to be a lot more work done with the government to buy into the work, own bits of it, and coordinate it again.”*

(Stakeholder 6)

*“It is responsibility of all the sub recipients to conduct sensitized trainings between stakeholders including health facilities and so in time I think there has been a mild decrease in stigma and discrimination towards sex workers in the last decade as a result of the sensitized trainings.”*

(Stakeholder 1)

Sub-theme 1.4: Community Engagement

Peer-led interventions have been instrumental in building trust and improving service uptake among FSWs. The DSD models programs empower sex workers to act as educators and advocates within their communities. Community-driven models that recruit peer educators from within the population foster trust, enhance service accessibility, and provide broader socio-economic benefits through skill development and employment.

*“Well, I think the South Africa sex work program has been a major success…peer educators who are sex workers are from the same area, location and venues…”*

(Stakeholder 1)

Theme 2: Sustainability and Innovation

Sustaining and improving healthcare services requires adequate funding, technological advancements, and efficient resource allocation. Increased government investment, donor engagement, and strategic partnerships are essential to ensure the sustainability and expansion of DSD programs. Without consistent funding and government ownership, these programs risk fragmentation.

Sub-theme 2.1: Funding and Resource Allocation

Sustainable funding remains a major challenge for DSD programs targeting KPs. Long-term sustainability demands increased government investment, strategic private sector partnerships, and sustained advocacy to secure adequate funding for KPs. Without coordinated efforts and government ownership, critical interventions risk fragmentation and inefficacy.

*“The next issue is that we are seeing 1% of the HIV budget goes to key pops…it is negligible…”*

(Stakeholder 1)

*“A lot more funding needs to be injected into stigma, discrimination, sensitizing, and training of police and healthcare workers…”*

(Stakeholder 6)

Sub-theme 2.2: Technology and Data Management

Technology has improved health care delivery and tracking among mobile KPs. Biometric systems and digital health records support accurate data management and adherence monitoring. Leveraging technology enhances continuity of care and data integrity. Biometric identifiers and electronic health records facilitate seamless service provision, particularly for highly mobile populations.

*“Sex worker program has a unique identifier…the biometric has been a game changer for adherence support…”*

(Stakeholder 1)

*“…we move into the digital world as a recommendation, like your electronic health records…you can go anywhere without your card or file but you can easily access anything you want with just your thumbs.”*

(Stakeholder 7)

Theme 3: Structural and systematic barriers

Key populations, particularly FSWs, face multifaceted barriers in accessing health care services. These barriers are embedded within structural, legal, and societal systems that collectively hinder access, adherence, and continuity of care.

Sub-theme 3.1: Access to Health care Services

Stakeholders identified challenges such as criminalization of sex work and insufficient sensitization among health care providers, which exacerbate the situation.

*“Criminalization of sex work interrupts in a negative way because if a particular location the law enforcement agents come and raid sex workers, they run away or we don’t know if the law enforcers took them, then there would be interruption of treatment or if there were 80 people, so it means 80 people in that area we won’t know where they are … then that is going to interrupt the services in that area.”*

(Stakeholder 8)

*“And if you were to look at the key population data in... South Africa, now that would still be a huge issue. We can talk to 95-95-95, but sex workers are incredibly mobile; they move from one place to another. The lack of trust issues and providing the right details, as well as the difficulty of de-duplicating across the program … means you can never really be sure if you have got an accurate number.”*

(Stakeholder 6)

*“I think what can work for the Department of Health is to generally sanitise their staff and then tailor the services to key populations to ensure that their programs and in that way one would tailor the services according to their needs”*

(Stakeholder 8)

Sub-theme 3.2: Criminalization

Criminalization fosters fear and mistrust, discouraging sex workers from engaging with healthcare services.

*“Generally one we need to recognize that the sex worker or sex work in the country is criminalized so, we need to be aware that we operating in a space where people are accessing our services so in that way it is sometimes difficult to always know that they will adhere to their appointments because maybe they would have some interaction with the law enforcement agency and make them to go hide or be taken.”*

(Stakeholder 8)

Sub-theme 3.3: Mobility and Continuity of Care

Mobility of FSWs poses additional challenges to maintain consistent health care engagement. As sex workers move for economic opportunities, continuity of care often suffers. Stakeholders stressed the importance of proactive communication and coordination:

*“We engage the sex workers that they must communicate, we cannot tell them that they should not travel or not move, but when they move they must communicate and take our numbers…”*

(Stakeholder 8)

*“…most of the time they do not communicate and in that way they might miss their treatment…they would go and work where the money is and then the continuity of care gets affected.”*

(Stakeholder 8)

Sub-theme 3.4: Stigma and Discrimination

Stigma, both societal and institutional, remains a pervasive barrier to health care access. Discriminatory attitudes by healthcare workers can deter FSWs from seeking services.

*“You can do the DSD model, but if stigma and discrimination are a huge issue there, it is more ingrained in the community and the space than other districts, and it is harder to implement.”*

(Stakeholder 6)

*“For the sex workers, once you bring the zero stigma, zero discrimination, then that is when you are able to engage more sex workers…if you use a human rights-based approach you are able to implement without any obstacles.”*

(Stakeholder 3)

Theme 4: Facilitators of DSD model implementation

Sub-theme 4.1: Decentralized community-based nature of DSD model

The decentralized, community-based nature of the DSD models brings services, such as ART and PrEP, closer to where FSWs live and work. Mobile clinics, adherence clubs, and pick-up points have helped bridge the gap between KPs and the formal health care system. Peer-led models have also proven instrumental, with trained sex workers acting as educators and support agents, increasing trust and improving adherence.

*“One of the huge successes was the building up of sex workers into peer educators, supporting them to have a job, to learn, to do something else, and giving them confidence and power often changed lives and helped them stay healthier, being on PrEP and being role models to their cohorts.”*

(Stakeholder 6)

*“Well, I think the SA sex work program has been a major success, from early on we were able to reach about 40k sex workers every quarter and the basic model would. I think the sex workers micro-planning, peer education lead is also part of DSD right, so even though the model has been refined over the last 15 years, essentially when I was still involved it was still the same idea behind, you have a cohort of peer educators who would, be sex workers and from the same area, location and venues, same gender, language, age so they were matched to the demographic of the sex workers they were to reach out and they know where the focus is.”*

(Stakeholder 1)

Sub-theme 4.2: Integration of clinical and psychosocial support

The integration of clinical and psychosocial support, particularly mental health services, has improved health outcomes and sustained engagement in care.

*“I think HIV program or DSD model should also involve the mental health so that clients can be motivated to continue to be on ARVs and then the other thing is as much as we are talking about testing, remember it is about the 95 95 95, we also need to ensure that those that have been tested are also linked to care and then continue with treatment…”*

(Stakeholder 5)

*“We have seen also that the viral loads were very high to the majority of the sex workers when we started. Then I included the social workers in the programme and we saw the viral load decreasing and majority of sex workers on treatment were virally suppressed.”*

(Stakeholder 8)

## 4. Discussion

This study illuminates the complex landscape of the DSD models implementation among FSWs in South Africa, revealing an array of both notable achievements and persistent challenges. At the heart of successful DSD implementation lies a collaborative, multi-faceted approach, demanding the concerted efforts of diverse stakeholders. Government departments and UN agencies emerge as pivotal actors, wielding considerable influence through policy formulation and technical guidance [13], thereby shaping the very foundation of these models. Equally significant are sex worker advocacy groups, whose relentless advocacy ensures that DSD strategies are meticulously tailored to address the unique and often marginalized realities of FSWs. Implementing partners translate policy into action, bridging the gap between theoretical frameworks and practical interventions. Local communities and health care providers, operating at the grassroots level, forge crucial bonds of trust, fostering engagement and ensuring the effectiveness of the models [10]. While international donors contribute essential financial resources, their engagement often lacks deep-seated interest in long-term community outcomes that characterizes local stakeholders transition planning [37].

The study identified key themes that underscore the successes and barriers encountered in the DSD implementation. Decentralized, community-based models, particularly those leveraging peer-led interventions, have proven remarkably effective in expanding access to HIV services [38]. The integration of clinical and psychosocial support has enhanced treatment adherence and overall well-being of FSWs [23,39]. Technological advancements, such as biometric systems and electronic health records, have streamlined patient tracking and bolstered care continuity [40]. However, the adaptability of the DSD models, while a strength, is continually tested by the complex needs of the FSWs [12,41,42].

Conversely, significant barriers impede the full realization of the DSD model potential. The criminalization of sex work casts a long shadow, engendering fear and mistrust that disrupt access to health care [43,44]. The high mobility of FSWs complicates treatment adherence, demanding flexible and responsive service delivery [45]. Pervasive stigma and discrimination, both within health care settings and the broader society, prevent service utilization among FSWs. Unsustainable funding and fragmented service delivery systems threaten the long-term viability of these programs [12,39]. Furthermore, insufficient sensitization of health care workers and law enforcement personnel undermines the establishment of trust and respect [42,46].

In addition to implementation successes, stakeholders expressed positive views about the adaptability of the DSD models. Its flexible structure allows health care services to be tailored to the unique needs of FSWs, including accommodating their mobility, irregular schedules, and experience with health care-related stigma. The expansion of HIV prevention services, such as pre-exposure prophylaxis (PrEP), STI treatment, and condom distribution within mobile clinics and outreach programs, reflects a trend seen in other African countries. In Tanzania, for example, differentiated models led to increased uptake of PrEP and a decline in STI incidence among FSWs [41]. Furthermore, South Africa’s own implementation of community ART delivery and adherence clubs has been linked with higher retention and viral suppression rates [12]. These findings reinforce the global consensus that DSD models must be tailored to local contexts and the specific needs of KPs to achieve optimal results [42].

However, the implementation of the DSD models for FSWs is not without significant barriers. Law enforcement actions, including raids and arrests, frequently disrupt service access and continuity of care. Stakeholders noted that criminalization drives sex workers underground, making it harder to reach them with consistent health care support. These insights are corroborated by other studies, which report that fear of arrest and police harassment deter sex workers from engaging with HIV services [43,44]. In addition, mobility among FSWs complicates continuity of care; frequent movement in search of work often leads to missed appointments and treatment interruptions. Studies from East and Southern Africa similarly note that high mobility disrupts follow-up care and undermines adherence [45].

Stigma and discrimination within health facilities remain a pervasive challenge, as highlighted by both stakeholders and previous research. Negative attitudes from health care workers reduce service engagement and lead to poor health outcomes [47]. Studies across sub-Saharan Africa have shown that health care provider stigma is a major barrier to care for sex workers, necessitating interventions such as structural competency training and rights-based sensitization [48]. Another persistent issue is the sustainability of DSD programs. Many remain heavily dependent on external donor funding, with limited domestic investment and sub-optimal government ownership. This trend mirrors findings from [49,50], who caution that, without domestic resource mobilization, many KPs programs risk collapse. Lastly, despite promising digital innovations, gaps in infrastructure, training and data security continue to hinder widespread adoption [45,46].

The implications of these findings are profound. Effective DSD implementation necessitates a collaborative ecosystem, where government, civil society, and international partners work in unison. Urgent policy reform, particularly the decriminalization of sex work, is essential to dismantle legal barriers to health care access. Health care delivery models must be agile and adaptable, accommodating the high mobility and unique needs of FSWs. Comprehensive interventions are required to address stigma and discrimination, encompassing community engagement and health care provider sensitization. Sustainable funding and enhanced coordination across service delivery systems are indispensable for long-term program success. Finally, ongoing sensitization and capacity building of health care workers and law enforcement personnel are vital to ensure the delivery of effective and respectful services.

The study underscores that, while the DSD models have demonstrated significant potential in improving access to HIV service for FSWs in South Africa, the realization of these potential hinges on dismantling systemic barriers, particularly the criminalization of sex work and its concomitant pervasive stigma. This requires a sustained, multi-faceted approach that prioritizes the health and dignity of one of the country’s most marginalized populations.

## 5. Conclusions

This study emphasizes the vital contribution of the DSD models in enhancing access to HIV prevention and treatment services among FSWs in South Africa. Interventions rooted in community outreach, peer-led engagement, and mobile healthcare have significantly extended service coverage, supported treatment adherence, and fostered trust between FSWs and the health care system. The integration of both clinical care and psychosocial support alongside the adoption of digital tools like biometric identification and electronic medical records have further strengthened service responsiveness to the unique needs of mobile and often marginalised populations.

Nevertheless, the implementation of DSD continues to face major structural, legal, and systemic obstacles. Chief among these are the criminalisation of sex work, entrenched stigma and discrimination, and the challenges associated with the high mobility of FSWs. These issues disrupt continuity of care and limit service utilisation. Therefore, there is a need for targeted efforts to sensitise health care workers and law enforcement to foster inclusive, respectful service environments.

The findings further highlight the importance of coordinated multi-sectoral collaboration in driving effective DSD implementation. Government entities and UN agencies play a key role in providing policy leadership and technical input, while sex worker-led organisations and implementing partners ensure services are context-sensitive and grounded in lived experiences. Peer-led, community-centred models are especially effective in increasing service relevance, acceptability, and reach.

To promote long-term sustainability and scale-up, stronger domestic investment, enhanced health infrastructure, and better cross-sectoral coordination are required. Reducing reliance on external donor support through local resource mobilisation and strategic public–private partnerships will be critical to maintaining progress. Moreover, the success of digital health tools depends on adequate infrastructure, skilled personnel, and secure data systems.

## 6. Strengths and Limitations

The stakeholders who participated in the study were well-versed in providing services to FSWs and other KPs, with extensive experience spanning the periods before, during, and after the COVID-19 lockdown period. The study provided insights into the implementation of the DSD models in South Africa, focusing on their roles, successes, challenges, and recommendations for enhancement. The study had a limited sample of single interview categories of stakeholders, who may not represent the views of majority in South Africa. The categories of the stakeholders were included to enable diverse, rich, and comprehensive information on the implementation of the HIV treatment DSD models. The inclusion of these diverse stakeholders enabled the harmonization of range of perspective, insights, experiences providing a comprehensive understanding of the DSD models, while limiting the influence of individual biases and allowing for diverse views of the stakeholders to be considered. However, these different categories might have limitations in terms of comparability across stakeholders. Future studies should focus on fewer categories of stakeholders to further elicit their roles and views on the implementation of the DSD models.

Therefore, regarding the generalizability of the findings, the study addresses this by offering a detailed and transparent account of the research methodology, enabling potential transferability to similar contexts. Due to USAID STOP orders in place at the time of data collection, not all targeted stakeholders could be reached, and some individuals were reluctant to participate, which further constrained the breadth of perspectives in the study.

## Figures and Tables

**Table 1:** Stakeholder mapping.

Stakeholder Type	Role/Interest/Contribution	Influence/Power/Impact Level
Government departments	Policy development, guidance, and implementation	High
UN Agency	Technical assistance and support	High
Sex Worker Advocacy Group	Advocacy and leadership	Medium-to-High
Implementing Partners	Advocacy and implementation	Medium

## Data Availability

The raw data that supports the findings of this study are available from the corresponding author, L.M., upon fair and reasonable request.

## Abbreviations

The following abbreviations are used in this manuscript:

AFSA	AIDS Foundation of South Africa
AIDS	Acquired Immunodeficiency Syndrome
ART	Antiretroviral Therapy
ARVs	Antiretroviral drugs
CBOs	Community-Based Organizations
CSOs	Civil Society Organizations
DoH	Department of Health
DSD	Differentiated Service Delivery
FSWs	Female Sex Workers
GF	Global Fund
HIV	Human Immunodeficiency Virus
KPs	Key Populations
MMD	Multi-Month Dispensing
NGOs	Non-Governmental Organizations
NDoH	National Department of Health
PEPFAR	President’s Emergency Plan for AIDS Relief
PLHIV	People Living with HIV
PrEP	Pre-Exposure Prophylaxis
REC	Research Ethics Committee
SANAC	South African National AIDS Council
STI(s)	Sexually Transmitted Infection(s)
UJ	University of Johannesburg
UN	United Nations
USAID	United States Agency for International Development
WHO	World Health Organization
